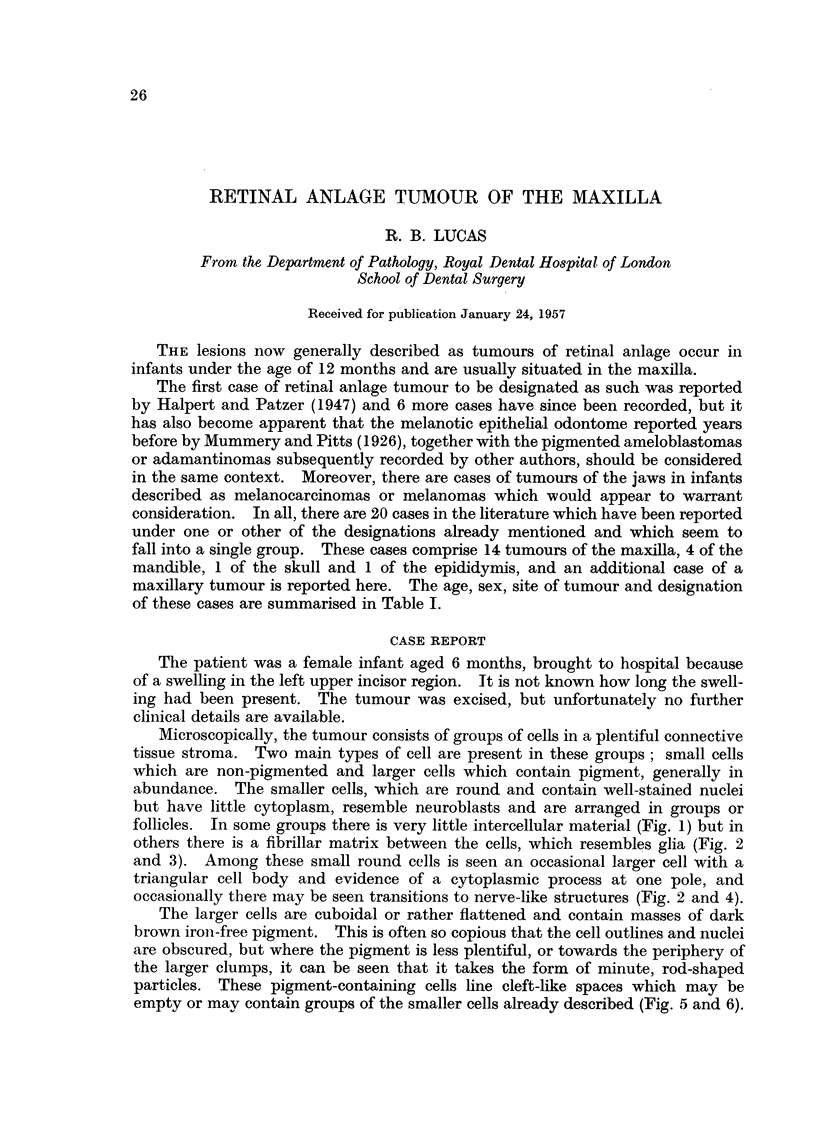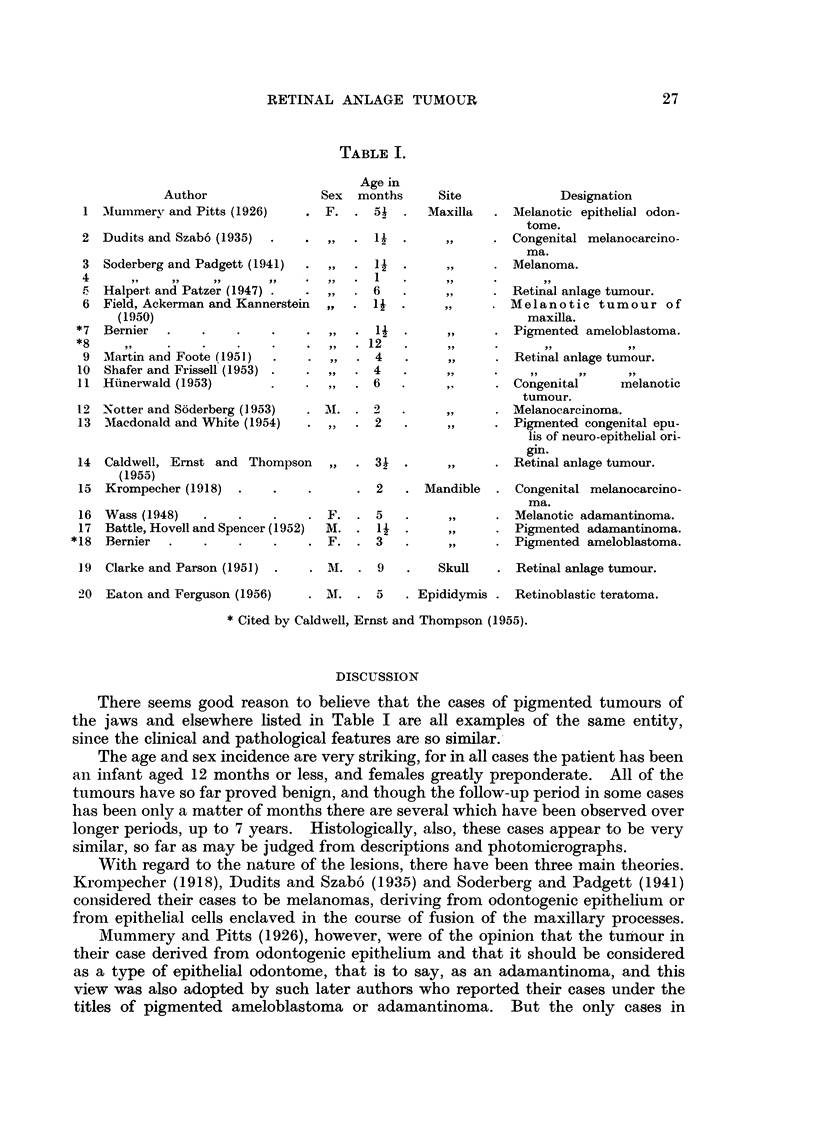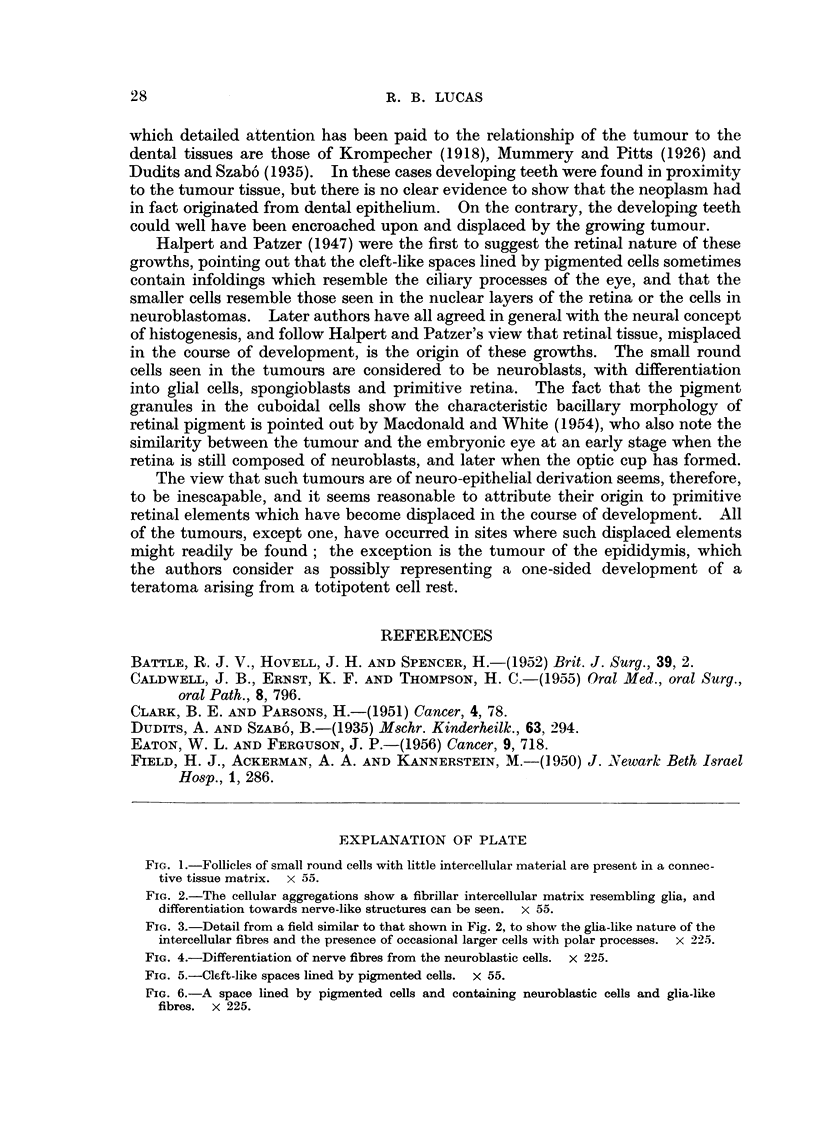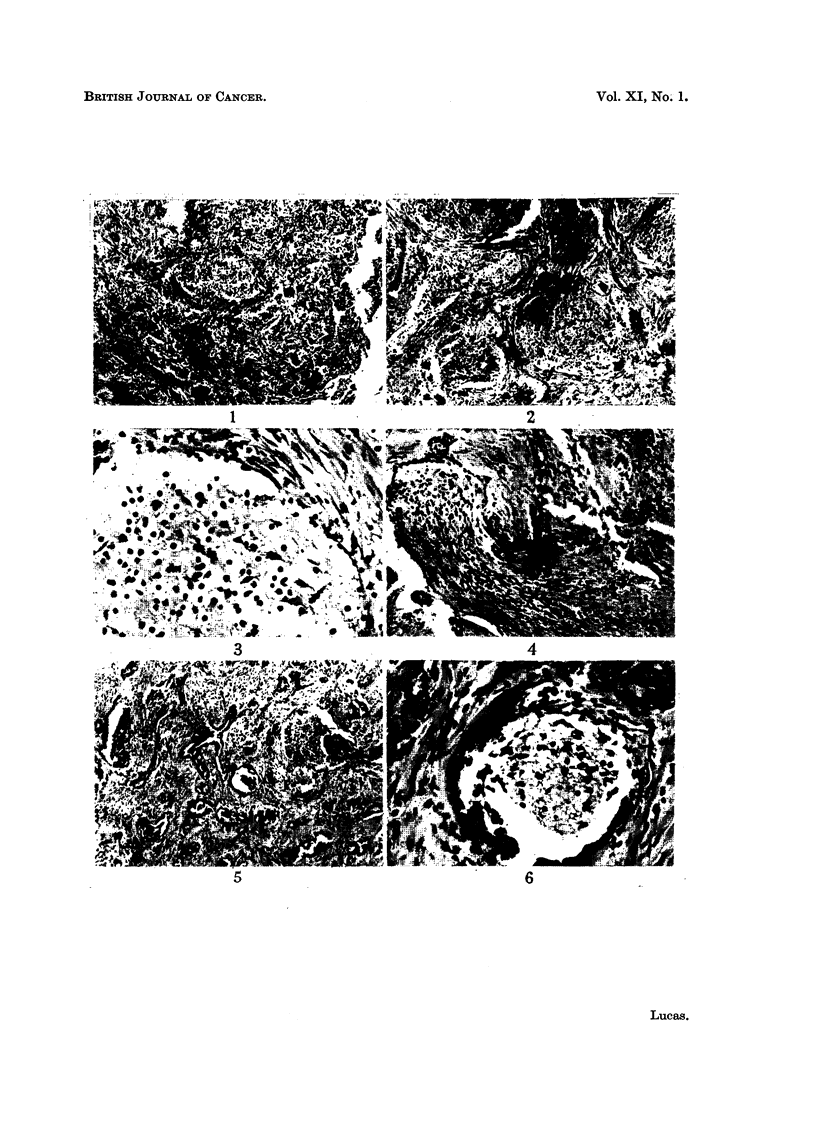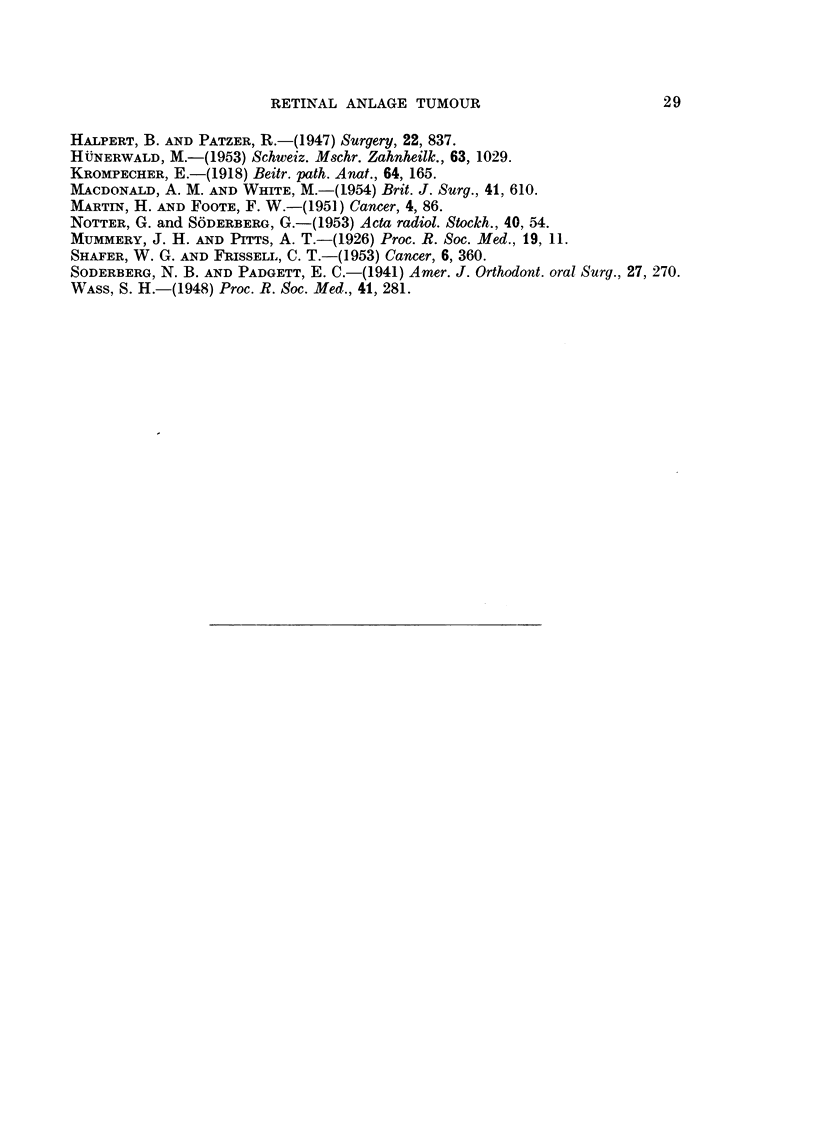# Retinal Anlage Tumour of the Maxilla

**DOI:** 10.1038/bjc.1957.4

**Published:** 1957-03

**Authors:** R. B. Lucas

## Abstract

**Images:**


### RETINAL ANLAGE TUMOUR OF THE MAXILLA

R. B. LUCAS

*From the Department of Pathology, Royal Dental Hospital of London School of Dental Surgery*

Received for publication January 24, 1957

THE lesions now generally described as tumours of retinal anlage occur in infants under the age of 12 months and are usually situated in the maxilla.

The first case of retinal anlage tumour to be designated as such was reported by Halpert and Patzer (1947) and 6 more cases have since been recorded, but it has also become apparent that the melanotic epithelial odontome reported years before by Mummery and Pitts (1926), together with the pigmented ameloblastomas or adamantinomas subsequently recorded by other authors, should be considered in the same context. Moreover, there are cases of tumours of the jaws in infants described as melanocarcinomas or melanomas which would appear to warrant consideration. In all, there are 20 cases in the literature which have been reported under one or other of the designations already mentioned and which seem to fall into a single group. These cases comprise 14 tumours of the maxilla, 4 of the mandible, 1 of the skull and 1 of the epididymis, and an additional case of a maxillary tumour is reported here. The age, sex, site of tumour and designation of these cases are summarised in Table I.

### CASE REPORT

The patient was a female infant aged 6 months, brought to hospital because of a swelling in the left upper incisor region. It is not known how long the swelling had been present. The tumour was excised, but unfortunately no further clinical details are available.

Microscopically, the tumour consists of groups of cells in a plentiful connective tissue stroma. Two main types of cell are present in these groups ; small cells which are non-pigmented and larger cells which contain pigment, generally in abundance. The smaller cells, which are round and contain well-stained nuclei but have little cytoplasm, resemble neuroblasts and are arranged in groups or follicles. In some groups there is very little intercellular material (Fig. 1) but in others there is a fibrillar matrix between the cells, which resembles glia (Fig. 2 and 3). Among these small round cells is seen an occasional larger cell with a triangular cell body and evidence of a cytoplasmic process at one pole, and occasionally there may be seen transitions to nerve-like structures (Fig. 2 and 4).

The larger cells are cuboidal or rather flattened and contain masses of dark brown iron-free pigment. This is often so copious that the cell outlines and nuclei are obscured, but where the pigment is less plentiful, or towards the periphery of the larger clumps, it can be seen that it takes the form of minute, rod-shaped particles. These pigment-containing cells line cleft-like spaces which may be empty or may contain groups of the smaller cells already described (Fig. 5 and 6).

TABLE I.

| | Author | Sex | Age in months | Site | Designation |
|---|---|---|---|---|---|
| 1 | Mummery and Pitts (1926) | F. | 5½ | Maxilla | Melanotic epithelial odontome. |
| 2 | Dudits and Szabó (1935) | ,, | 1½ | ,, | Congenital melanocarcinoma. |
| 3 | Soderberg and Padgett (1941) | ,, | 1½ | ,, | Melanoma. |
| 4 | ,, ,, ,, ,, | ,, | 1 | ,, | ,, |
| 5 | Halpert and Patzer (1947) | ,, | 6 | ,, | Retinal anlage tumour. |
| 6 | Field, Ackerman and Kannerstein (1950) | ,, | 1½ | ,, | Melanotic tumour of maxilla. |
| *7 | Bernier | ,, | 1½ | ,, | Pigmented ameloblastoma. |
| *8 | ,, | ,, | 12 | ,, | ,, ,, |
| 9 | Martin and Foote (1951) | ,, | 4 | ,, | Retinal anlage tumour. |
| 10 | Shafer and Frissell (1953) | ,, | 4 | ,, | ,, ,, ,, |
| 11 | Hünerwald (1953) | ,, | 6 | ,, | Congenital melanotic tumour. |
| 12 | Notter and Söderberg (1953) | M. | 2 | ,, | Melanocarcinoma. |
| 13 | Macdonald and White (1954) | ,, | 2 | ,, | Pigmented congenital epulis of neuro-epithelial origin. |
| 14 | Caldwell, Ernst and Thompson (1955) | ,, | 3½ | ,, | Retinal anlage tumour. |
| 15 | Krompecher (1918) | | 2 | Mandible | Congenital melanocarcinoma. |
| 16 | Wass (1948) | F. | 5 | ,, | Melanotic adamantinoma. |
| 17 | Battle, Hovell and Spencer (1952) | M. | 1½ | ,, | Pigmented adamantinoma. |
| *18 | Bernier | F. | 3 | ,, | Pigmented ameloblastoma. |
| 19 | Clarke and Parson (1951) | M. | 9 | Skull | Retinal anlage tumour. |
| 20 | Eaton and Ferguson (1956) | M. | 5 | Epididymis | Retinoblastic teratoma. |

* Cited by Caldwell, Ernst and Thompson (1955).

### DISCUSSION

There seems good reason to believe that the cases of pigmented tumours of the jaws and elsewhere listed in Table I are all examples of the same entity, since the clinical and pathological features are so similar.

The age and sex incidence are very striking, for in all cases the patient has been an infant aged 12 months or less, and females greatly preponderate. All of the tumours have so far proved benign, and though the follow-up period in some cases has been only a matter of months there are several which have been observed over longer periods, up to 7 years. Histologically, also, these cases appear to be very similar, so far as may be judged from descriptions and photomicrographs.

With regard to the nature of the lesions, there have been three main theories. Krompecher (1918), Dudits and Szabó (1935) and Soderberg and Padgett (1941) considered their cases to be melanomas, deriving from odontogenic epithelium or from epithelial cells enclaved in the course of fusion of the maxillary processes.

Mummery and Pitts (1926), however, were of the opinion that the tumour in their case derived from odontogenic epithelium and that it should be considered as a type of epithelial odontome, that is to say, as an adamantinoma, and this view was also adopted by such later authors who reported their cases under the titles of pigmented ameloblastoma or adamantinoma. But the only cases in which detailed attention has been paid to the relationship of the tumour to the dental tissues are those of Krompecher (1918), Mummery and Pitts (1926) and Dudits and Szabó (1935). In these cases developing teeth were found in proximity to the tumour tissue, but there is no clear evidence to show that the neoplasm had in fact originated from dental epithelium. On the contrary, the developing teeth could well have been encroached upon and displaced by the growing tumour.

Halpert and Patzer (1947) were the first to suggest the retinal nature of these growths, pointing out that the cleft-like spaces lined by pigmented cells sometimes contain infoldings which resemble the ciliary processes of the eye, and that the smaller cells resemble those seen in the nuclear layers of the retina or the cells in neuroblastomas. Later authors have all agreed in general with the neural concept of histogenesis, and follow Halpert and Patzer's view that retinal tissue, misplaced in the course of development, is the origin of these growths. The small round cells seen in the tumours are considered to be neuroblasts, with differentiation into glial cells, spongioblasts and primitive retina. The fact that the pigment granules in the cuboidal cells show the characteristic bacillary morphology of retinal pigment is pointed out by Macdonald and White (1954), who also note the similarity between the tumour and the embryonic eye at an early stage when the retina is still composed of neuroblasts, and later when the optic cup has formed.

The view that such tumours are of neuro-epithelial derivation seems, therefore, to be inescapable, and it seems reasonable to attribute their origin to primitive retinal elements which have become displaced in the course of development. All of the tumours, except one, have occurred in sites where such displaced elements might readily be found ; the exception is the tumour of the epididymis, which the authors consider as possibly representing a one-sided development of a teratoma arising from a totipotent cell rest.

### REFERENCES

Battle, R. J. V., Hovell, J. H. and Spencer, H.—(1952) *Brit. J. Surg.*, **39**, 2.

Caldwell, J. B., Ernst, K. F. and Thompson, H. C.—(1955) *Oral Med., oral Surg., oral Path.*, **8**, 796.

Clark, B. E. and Parsons, H.—(1951) *Cancer*, **4**, 78.

Dudits, A. and Szabó, B.—(1935) *Mschr. Kinderheilk.*, **63**, 294.

Eaton, W. L. and Ferguson, J. P.—(1956) *Cancer*, **9**, 718.

Field, H. J., Ackerman, A. A. and Kannerstein, M.—(1950) *J. Newark Beth Israel Hosp.*, **1**, 286.

---

### EXPLANATION OF PLATE

Fig. 1.—Follicles of small round cells with little intercellular material are present in a connective tissue matrix. × 55.

Fig. 2.—The cellular aggregations show a fibrillar intercellular matrix resembling glia, and differentiation towards nerve-like structures can be seen. × 55.

Fig. 3.—Detail from a field similar to that shown in Fig. 2, to show the glia-like nature of the intercellular fibres and the presence of occasional larger cells with polar processes. × 225.

Fig. 4.—Differentiation of nerve fibres from the neuroblastic cells. × 225.

Fig. 5.—Cleft-like spaces lined by pigmented cells. × 55.

Fig. 6.—A space lined by pigmented cells and containing neuroblastic cells and glia-like fibres. × 225.

1 2

3 4

5 6

HALPERT, B. AND PATZER, R.—(1947) *Surgery*, **22**, 837.
HÜNERWALD, M.—(1953) *Schweiz. Mschr. Zahnheilk.*, **63**, 1029.
KROMPECHER, E.—(1918) *Beitr. path. Anat.*, **64**, 165.
MACDONALD, A. M. AND WHITE, M.—(1954) *Brit. J. Surg.*, **41**, 610.
MARTIN, H. AND FOOTE, F. W.—(1951) *Cancer*, **4**, 86.
NOTTER, G. and SÖDERBERG, G.—(1953) *Acta radiol. Stockh.*, **40**, 54.
MUMMERY, J. H. AND PITTS, A. T.—(1926) *Proc. R. Soc. Med.*, **19**, 11.
SHAFER, W. G. AND FRISSELL, C. T.—(1953) *Cancer*, **6**, 360.
SODERBERG, N. B. AND PADGETT, E. C.—(1941) *Amer. J. Orthodont. oral Surg.*, **27**, 270.
WASS, S. H.—(1948) *Proc. R. Soc. Med.*, **41**, 281.